# MicroRNA‐638 inhibits human aortic valve interstitial cell calcification by targeting Sp7

**DOI:** 10.1111/jcmm.14405

**Published:** 2019-05-29

**Authors:** Wenjie Jiao, Dongyang Zhang, Dong Wang, Rongwei Xu, Linna Tang, Min Zhao, Rongjian Xu

**Affiliations:** ^1^ Department of Thoracic Surgery The Affiliated Hospital of Qingdao University Qingdao China; ^2^ Department of Vascular Surgery Shandong Provincial Qianfoshan Hospital, Shandong University Jinan China; ^3^ Department of Hospital Infection Control Shandong Provincial Qianfoshan Hospital, Shandong University Jinan China; ^4^ Center of Laboratory Medicine Qilu Hospital of Shandong University (Qingdao) Qingdao China

**Keywords:** calcific aortic valve disease, human aortic valve interstitial cells, miRNA‐638, osteogenic differentiation, Sp7

## Abstract

Calcific aortic valve disease (CAVD) is a complex heart valve disease involving a wide range of pathological changes. Emerging evidence indicates that osteogenic differentiation of human aortic valve interstitial cells (hAVICs) plays a key role in valve calcification. In this study, we aimed to investigate the function of miR‐638 in hAVICs osteogenesis. Both miRNA microarray assay and qRT‐PCR results demonstrating miR‐638 was obviously up‐regulated in calcific aortic valves compared with non‐calcific valves. We also proved that miR‐638 was significantly up‐regulated during hAVICs osteogenic differentiation. Overexpression of miR‐638 suppressed osteogenic differentiation of hAVICs in vitro, whereas down‐regulation of miR‐638 enhance the process. Target prediction analysis and dual‐luciferase reporter assay confirmed that Sp7 transcription factor (Sp7) was a direct target of miR‐638. Furthermore, knockdown of Sp7 inhibited osteogenic differentiation of hAVICs, which is similar to the results observed in up‐regulation miR‐638. Our data indicated that miR‐638 plays an inhibitory role in hAVICs osteogenic differentiation, which may act by targeting Sp7. MiR‐638 may be a potential therapeutic target for CAVD.

## 1 INTRODUCTION

Calcific aortic valve disease (CAVD), previously conceptualized as a passive and degenerative valve disorder, is now proved to be a complex and active pathological process characterized by osteogenesis, endothelial injury, inflammatory infiltration and matrix turnover.^1^, ^2^, ^3^, ^4^, ^5^, ^6^, ^7^ With the development of society and the prolongation of life span, the prevalence of CAVD has risen in recent years. At present, CAVD has become a major health issue threatening ageing population. Severe CAVD may result in devastating end‐stage cardiovascular dysfunction and even sudden death.^8^, ^9^ To date, there are still no effective clinical interventions to reverse CAVD or halt the progression. The only choice for treatment is cardiac surgery with implantation of a valve prosthesis.^10^ Therefore, it is critical to explore the molecular mechanisms by which aortic valve leaflets become calcified.

Calcific aortic valve disease has been the most common valve disease in the developed world. It is the third leading cause to cardiovascular diseases.^11^ Valve calcification is the typical characteristic of CAVD, with accumulating evidence for heterotopic ossification. Mohler et al confirmed that heterotopic ossification consisting of mature lamellar bone formation and active bone remodelling is relatively common in end‐stage calcific aortic valves.^12^ Studies had demonstrated the presence of osteoblast‐like cells, osteoblast‐specific transcripts and osteogenic markers in calcified aortic valves.^13^, ^14^ In addition, extracellular bone matrix proteins, such as osteopontin, osteonectin and bone morphogenetic proteins (BMPs) that are believed to play a part in the calcific process, are also found in calcific aortic valves.^12^, ^15^, ^16^, ^17^ These above results suggested that cellular osteogenic transdifferentiation may be correlated with the pathogenesis of CAVD.

The human aortic valves are composed of three small collagenous leaflets attached to the fibrous ring of the left ventricular outflow tract. The leaflets are composed of a dense extracellular matrix, usually divided into three layers: lamina fibrosa, lamina spongiosa and lamina ventricularis. All three layers are populated with aortic valve interstitial cells (AVICs), with the whole structure covered by a confluent monolayer of valve endothelial cells.^10^, ^18^ AVICs are the most predominant cells in aortic valves and play a key role in maintaining normal aortic valve structure and function.^19^ AVICs are capable of undergoing a phenotypic transition to become osteoblast‐like cells and elaborate bone matrix in response to osteogenic inductors during CAVD.^1^, ^20^, ^21^ Multiple studies have also showed that osteogenic differentiation of AVICs is involved in the pathogenesis of CAVD.^22^, ^23^, ^24^

MicroRNAs (MiRNAs) are small, single‐stranded, non‐coding RNAs that act as fine‐tuners in the negative regulation of gene expression by binding to complementary sequences in the 3‐untranslated region of targeted mRNA, thereby leading to either mRNA degradation or translational repression.^25^, ^26^, ^27^ MiRNAs are involved in diverse physiological and pathological processes, including cell development, proliferation, apoptosis and differentiation.^28^, ^29^, ^30^ Multiple miRNAs that regulate the process of osteogenic differentiation have been identified. For instance miRNA‐214 promotes periodontal ligament stem cell osteoblastic differentiation by modulating Wnt/β‐catenin signalling,^31^ whereas miRNA‐98 targets BMP‐2 to inhibit osteogenic differentiation of human bone mesenchymal stromal cells.^32^ Moreover, miRNA‐22 is confirmed to enhance osteogenic differentiation and inhibit adipogenic differentiation of human adipose tissue‐derived mesenchymal stromal cells by repressing histone deacetylase (HDAC6).^33^

In our preliminary study, we found that miRNA‐638 was significantly up‐regulated in calcific aortic valves compared with non‐calcific valves.^1^ In the present research, we further investigated the function of miRNA‐638 in osteogenic differentiation of hAVICs and identified target genes of miRNA‐638. Our findings indicate that miRNA‐638, which is up‐regulated in osteogenic differentiation of hAVICs, inhibits the differentiation process by repressing Sp7.

## 2 MATERIALS AND METHODS

### 2.1 Ethics statements

The study protocol was approved by the Ethical Committee of the Affiliated Hospital of Qingdao University, and informed consents were obtained from the human donors. All experiments were performed in accordance with the relevant guidelines and regulations.

### 2.2 Calcific aortic valve collection

Samples were obtained from 10 CAVD patients (Table 1), who had undergone cardiac surgery with implantation of a valve prosthesis. Exclusion criteria included non‐stenotic, congenital aortic valve disease, rheumatic aortic valve disease, genetic disease and autoimmune disease. During the operation, two tissue Samples were taken from each patient for the follow‐up study: one was calcific aortic valves that contained calcific nodules, and the other that serves as a control was adjacent non‐calcific aortic valve tissues surrounding calcific valves of the same patient. All samples were resected during the operation and immediately placed in pairs in liquid nitrogen for the following research. At the same time, pathological examinations of tissue samples from 10 patients were performed to make sure the accuracy of tissues sampling and trimming.

**Table 1:** Demographic characteristics of the patients (n = 10)

Parameters	Value
Age (y, mean ± SD)	67.2 ± 3.46
Sex ratio (male/female)	5:5
Reason for aortic valve replacement (no.)	
Valve stenosis	6
Valve stenosis and insufficiency	4
Systemic disease (no.)	
Diabetes (Type 2)	2
Hypertension	2

### 2.3 Microarray analysis

Calcific aortic valves and non‐calcific valves from three CAVD patients were sent to carry on the miRNA microarray assay. Total RNA was extracted from tissues using the miRNAeasy Mini Kit (Qiagen GmbH). The miRNA microarray assay was performed by a service provider (LC Sciences). Total RNA (100 ng) was labelled with miRNA Complete Labeling and Hyb Kit (Agilent, USA) and hybridized on the Human miRNA Microarray Kit (Release 16.0, Agilent), which contains 60 000 probes for 1205 and 144 human viral miRNAs. Hybridization signals were detected with the Agilent Microarray Scanner (Agilent, USA) and the scanned images were analysed using Agilent Feature Extraction Software (Agilent, USA). Data were acquired by first subtracting the background noise of raw data from hybridization images and then normalizing using the LOWESS filter (locally weighted regression).^34^ Spot (standard deviation)/(signal intensity) < 0.5. Differentially expressed miRNAs were identified by a cut‐off of fold change >1.5 and *P* < 0.01 by Student’s *t* test.

### 2.4 MiRNA real‐time quantitative PCR

MiRNA‐638 was extracted using the miRVana extraction kit (Ambion). For miRNA‐638 quantification, 10 ng total RNA was transcribed reversely and amplified using the miRNA reverse transcription and detection kit (Applied Biosystems, Inc). All results were normalized to U6 levels, which were determined by the ABI miRNA U6 assay kit (Applied Biosystems, Inc).

### 2.5 hAVICs isolation and cell culture

Normal aortic valves (n = 5) were derived from patients who had undergone acute Stanford A aortic dissection. Primary hAVICs were prepared as described previously.^1^, ^9^, ^35^ In brief, non‐leaflet tissues were carefully eliminated after effective removal of the endothelial layer of aortic and ventricular aspects, then valves were immersed in 0.25% trypsin at 37°C for 5 minutes. The tissues were then cut into pieces and digested for an additional 2 hours at 37°C. Primary hAVICs were obtained and seeded in growth medium (Dulbecco’s Modified Eagle Medium supplemented with penicillin and streptomycin, mem non‐essential amino acid, sodium pyruvate and 10% FBS) at 37°C under a 5% carbon dioxide atmosphere. The purity of hAVICs was confirmed by microscopic examination and evaluation of expression of marker proteins.

### 2.6 Transient transfection and cell treatments

Synthetic miRNA‐638 mimic (M‐miR‐638), miRNA‐638 inhibitor (I‐miR‐638), mimic and inhibitor negative controls (miR‐NC and miR‐NCI) and Sp7 siRNA (Si‐Sp7), were purchased from Guangzhou RiboBio Co., Ltd (China). hAVICs were seeded at a density of 3 × 10^6^ cells in 6‐well plates (Corning Costar, USA). When cells reached 70%‐80% confluence, hAVICs were individually transfected at a final concentration of 200 nmol/L in OPTI‐MEMI reduced serum medium (Invitrogen, USA) using lipofectamine 2000 (Invitrogen) according to the manufacturer’s instructions. Transfection efficiency was measure at day 3 in a preliminary test. Osteogenic differentiation was subsequently induced after transfection by culturing cells in osteogenic differentiation medium (growth medium supplemented with 500‐ng/mL BMP‐2, 100‐nmol/L dexamethasone, 50‐µg/mL ascorbic acid and 10‐mmol/L β‐glycerophosphate).

### 2.7 mRNA quantitative real‐time PCR

The mRNA expression of alkaline phosphatase (ALP), integrin binding sialoprotein (IBSP) and Sp7 were detected using qRT‐PCR after osteogenic induction of hAVICs. Total RNA was extracted with TRIzol reagent (Invitrogen). Power SYBR Green RT‐PCR Kit (Invitrogen) and Bio‐RAD CFX96 Real‐Time System (Bio‐Rad, USA) were used for quantitative RT‐PCR analysis. Data were normalized to the reference gene glyceraldehyde‐3‐phosphate dehydrogenase (GAPDH) for each cDNA sample. All primers used were synthetized by Sangon Biotech (China) and listed in Table 2.

**Table 2:** Primers used in qRT‐PCR

Gene	Primer sequences
Sp7	Forward: TCCCTTTTCCCACTCATTCC
	Reverse: GGGCAGACAGTCAGAAGAGC
ALP	Forward: CCACGTCTTCACATTTGGTG
	Reverse: AGACTGCGCCTGGTAGTTGT
IBSP	Forward: TGGATGAAAACGAACAAGGCA
	Reverse: AAACCCACCATTTGGAGAGGT
GAPDH	Forward: AGCCACATCGCTCAGACAC
	Reverse: TGGACTCCACGACGTACTC
miR‐638	Forward: GAGAGGATCCTGCCGCAGATCGCTG
	Reverse: GAGTAAGCTTCAGGGAGTCCTCTGCC
U6	Forward: CTCGCTCGGCAGAACA
	Reverse: AACGCTTCACGAATTTGCGT
Si‐Sp7	GGCAAAGCAGGCACAAAGA
Sp7 3UTR WT	Forward: TACTCAGAGCTCACTTTCTATTTGGGCTCCCAA
	Reverse: TACTCATCTAGAGACTTCATTACAAGAGAAACCCT
Sp7 3UTR MT	Forward: TACTCAGAGCTCACTTTCTATTTGGGCTCCCAA
	Reverse: TGGGGTCACCCCATCTTTATTCGTAGATCCCCACTGGTC
	Forward: GACCAGTGGGGATCTACGAATAAAGATGGGGTGACCCCA
	Reverse: TACTCATCTAGAGACTTCATTACAAGAGAAACCCT

### 2.8 Western blotting

The protein expression of ALP, IBSP and Sp7 were measured by using western blotting after osteogenic differentiation of hAVICs. The transfected hAVICs samples were fixed in 4% paraformaldehyde for 30 minutes, and then blocked with 0.2% Triton X‐100 and 3% goat serum in PBS. Cell lysate was separated by 12% sodium dodecyl sulphate‐polyacrylamide gelelectrophoresis (SDS‐PAGE) gel. Primary antibodies including anti‐Sp7 (Abcam, USA), anti‐ALP (Abcam), anti‐IBSP (Abcam) and anti‐GAPDH (Abcam) were incubated overnight at 4°C. After washing, membranes were incubated with secondary anti‐rabbit horseradish peroxidase‐conjugated antibodies (Elabscience Biotechnology Co., Ltd, China) for 2 hours at room temperature.

### 2.9 Dual luciferase reporter assay

The 3UTR of human gene Sp7 was amplified from human cDNA. The wide‐type fragment containing the predicted miRNA‐638 binding site and its mutant fragment, designed to carry sites for SacI (5 end) and XbaI (3 end) at their ends, were obtained from 3UTR of Sp7. Amplicons were cleaved with SacI and XbaI and inserted between SacI and XbaI cleavage sites of pmirGLO vector (Promega, USA). 293T cells were selected on the basis of the low endogenous miRNA expression. Cells were seeded in 24‐well plates. When it reached to 70%‐80% confluence, the 800 ng wild‐type or mutant reporter and 20 µmol/L M‐miR‐638, I‐miR‐638 or miR‐NC were cotransfected into 293T cells using Lipofectamine 2000 (Invitrogen). 24 hours after transfection, firefly and renilla luciferase activities were measured in cell lysates using the dual‐luciferase reporter system (%, USA).

### 2.10 Alkaline phosphatase activity assay

The osteogenic phenotype was determined based on the ALP activity, which is an early osteoblastic differentiation marker. The ALP activity assay was conducted on day 3 or 7 after osteogenic differentiation of hAVICs. Cells were washed twice with phosphate‐buffered saline solution (PBS) and lysed with 150 µL NP‐40 lysis buffer (Beyotime, China). The cell lysates were quantified by an alkaline phosphatase assay kit (Beyotime) using p‐nitrophenyl phosphate (pNPP) as the substrate. In the presence of magnesium ions, pNPP was hydrolysed by phosphatases to phosphate and p‐nitrophenol. The rate of p‐nitrophenol liberation is proportional to the ALP activity and can be measured photometrically. The ALP activity was measured by spectrophotometer at 405 nm.

### 2.11 Statistical analysis

Each experiment was repeated in triplicate at least three times. Statistical analysis which was performed by using SPSS 16.0. Data were presented as mean ± SD. Comparisons of parameters between two groups were evaluated by Students’ *t* test. Comparisons of parameters among more than two groups were analysed by one‐way ANOVA, and comparisons of different parameters between each group were made by a post hoc analysis using a Bonferroni’s test. Non‐parametric Mann‐Whitney *U* and Kruskal‐Wallis tests were performed when the sample size was smaller. Differences at *P* < 0.05 were considered to be statistically significant.

## 3 RESULTS

### 3.1 Expression level of miRNA‐638 is up‐regulated in human calcific aortic valves

**Figure 1:**
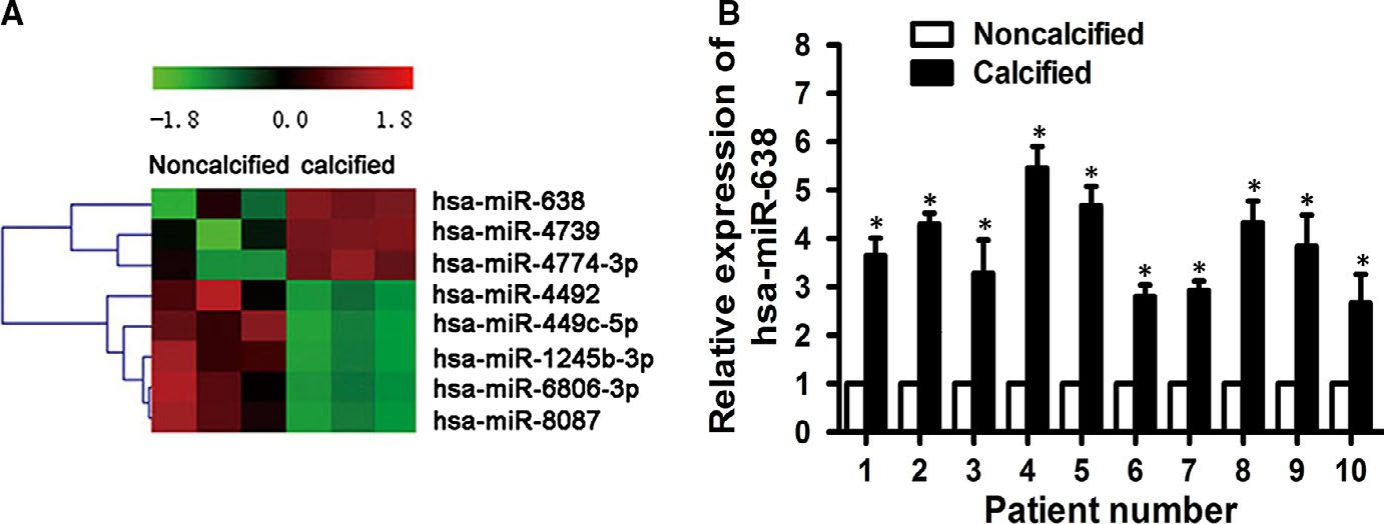
miRNA‐638 is up‐regulated in human calcific aortic valves. A, A heat map based on differentially expressed miRNAs between calcific and non‐calcific aortic valves calculated by microarray (n = 3). B, qRT‐PCR confirmation of expression level in calcific aortic valves from CAVD patients (n = 10). Data were presented as the mean ± SD. *P < 0.05

In order to identify the dysregulated miRNAs in CAVD pathogenesis, miRNA microarray assay was conducted to analyse the expression profile of miRNAs in non‐calcific and calcific aortic valves. A total of eight miRNAs was ultimately identified, including three up‐regulated miRNAs (miRNA‐638, miRNA‐4739, miRNA‐4774‐3p) and five down‐regulated miRNAs (miRNA‐4492, miRNA‐449c‐5p, miRNA‐1245‐3p, miRNA‐6806‐3p, miRNA‐8087) (Figure 1A).^1^ Then target gene prediction of these miRNAs was performed using miRNA databases (TargetScan 7.2). Interestingly, one of the predicted target genes of miRNA‐638 is Sp7 which is a pivotal transcription factor associated with osteogenic differentiation.^36^, ^37^ Thus, miRNA‐638 was chosen for further research in this study.

To investigate the accuracy of microarray result, miRNA‐638 expression of aortic valve tissues was detected using qRT‐PCR. We examined expression level of miRNA‐638 in the same set of 10 pairs of surgically resected calcific aortic valves and their adjacent non‐calcific valves. Our results showed that miRNA‐638 expression was significantly up‐regulated in calcific aortic valves compared to that of non‐calcific valves (Figure 1B), which suggested that miRNA‐638 might participate in the pathogenesis of CAVD.

### 3.2 Primary culture and phenotype identification of hAVICs

**Figure 2:**
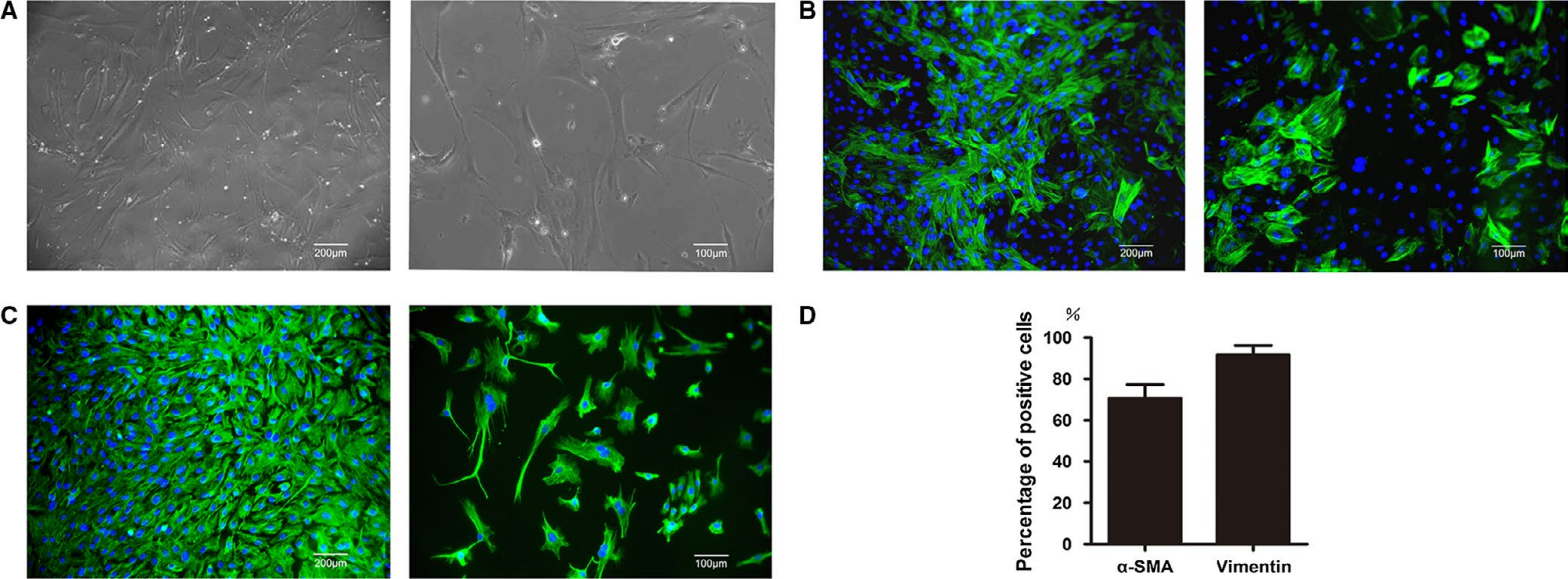
Characteristics and phenotype identification of hAVICs. A, The morphology of hAVICs. B, Immunohistochemical staining of α‐SMA. C, Immunohistochemical staining of vimentin. D, Quantification of positive staining of α‐SMA and vimentin. Left: low magnification; Right: high magnification

The primary hAVICs began to grow with adherence at about 2 days of primary culture. After 6 days, the adherent cells were flat and spindle‐shaped. The cells grew slowly and reached to about 70% confluences after 14 days of culture. During subsequent cell passages, the cell density was high and the cells were swirling and radial arranged (Figure 2A).

To further verify the isolated cells were hAVICs, two marker proteins associated with hAVICs were detected by immunohistochemical staining. The results showed hAVICs from three passages were positive for vimentin and α‐SMA (91% and 71% respectively) (Figure 2B‐D).

### 3.3 Expression level of miRNA‐638 increases during osteogenic differentiation of hAVICs

**Figure 3:**
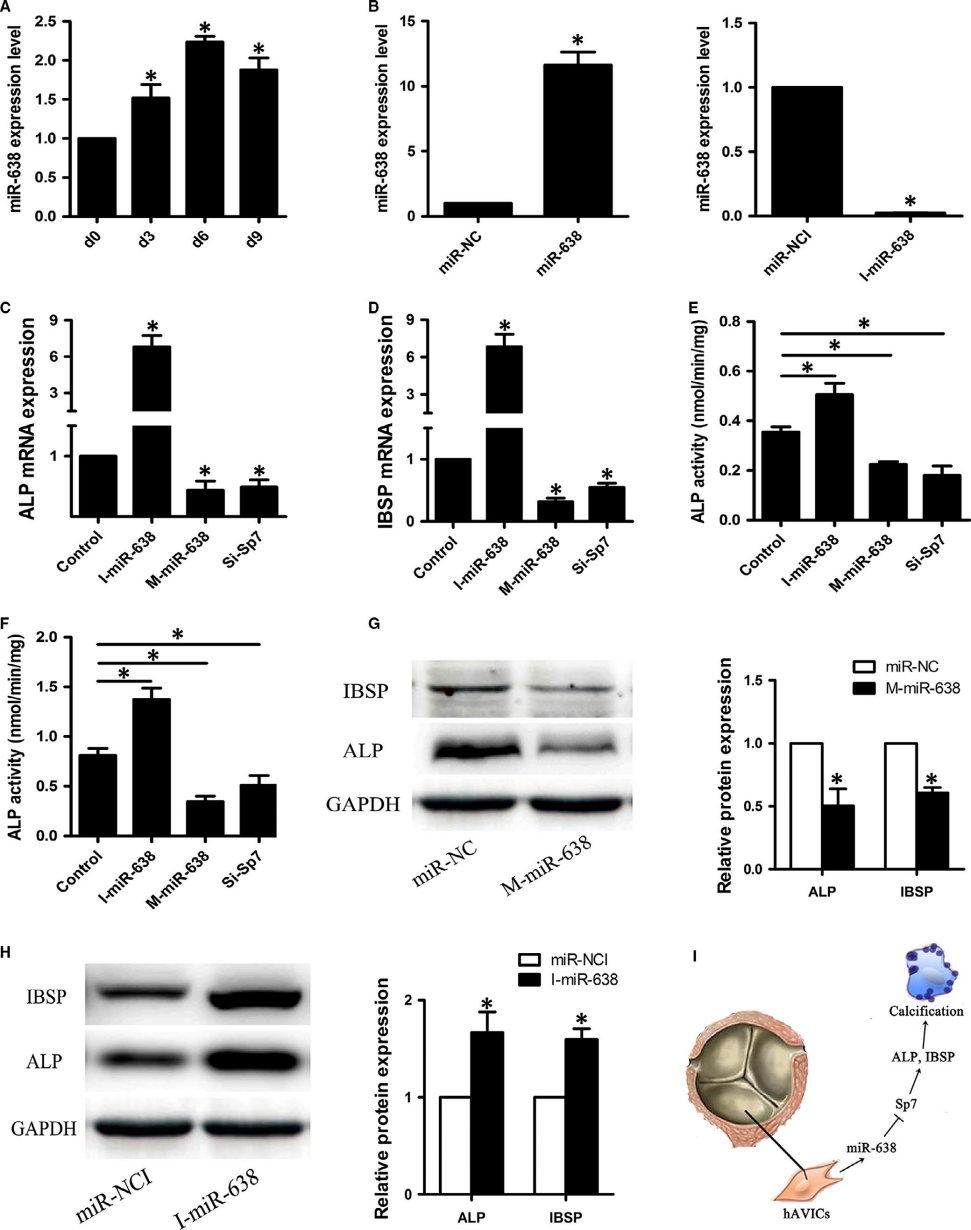
Overexpression of miRNA‐638 inhibits osteogenic differentiation of hAVICs, whereas downexpression of miRNA‐638 promotes the process. A, The endogenous expression level of miRNA‐638 was measured by qRT‐PCR at different time points during osteogenic differentiation (0, 3, 6 and 9 days) of hAVICs. The data, normalized to U6, are averages of three independent experiments (mean ± SD). *P < 0.05 versus day 0. B, The expression of miRNA‐638 in hAVICs transfected with miRNA mimic or inhibitor at day 3 was determined by qRT‐PCR compared with miR‐NC or miR‐NCI group. The data, normalized to U6, are averages of three independent experiments (mean ± SD). *P < 0.05 versus miR‐NC or miR‐NCI group. C and D, The mRNA expression of osteogenic specific genes ALP and IBSP after miRNA‐638 overexpression or downexpression at day 7 was analysed by qRT‐PCR. The data, normalized to GAPDH, are averages of three independent experiments (mean ± SD). *P < 0.05. E, ALP activity at day 3 after osteogenic differentiation of hAVICs was measure in each group. The data are averages of three independent experiments (mean ± SD). *P < 0.05. F, ALP activity at day 7 after osteogenic differentiation of hAVICs was measure in each group. The data are averages of three independent experiments (mean ± SD). *P < 0.05. G and H, Western blotting results showed the protein expression of ALP and IBSP at day 14 after osteogenic differentiation of hAVICs transfected with miRNA‐638 mimic or inhibitor. The data, normalized to GAPDH, are averages of three independent experiments (mean ± SD). *P < 0.05 versus miR‐NC or miR‐NCI group. I, Schematic diagram depicting the mechanism underlying aortic valve calcification in this study. ‘┥’: inhibit, ‘→’: promote

To explore the role of miRNA‐638 in osteogenic differentiation of hAVICs, these cells were harvested at different time points in the process of osteogenic differentiation (0, 3, 6 and 9 days), and miRNA‐638 expression level was analysed by qRT‐PCR. Expression of miRNA‐638 increased on day 3 compared with that of untreated control hAVICs (day 0) and remained high until day 9 (Figure 3A). This result indicates that miRNA‐638 might negatively regulate osteogenic differentiation of hAVICs.

### 3.4 miRNA‐638 inhibits hAVICs osteogenic differentiation

To further clarify the function of miRNA‐638 in regulation of osteogenic differentiation, synthetic mimic and inhibitor of miRNA‐638 were transfected into hAVICs, and osteogenic capacity was detected by ALP activity, qRT‐PCR and western blotting. First, efficiency of miRNA‐638 transfection was estimated by qRT‐PCR. Intracellular miRNA‐638 levels were significantly up‐regulated by miRNA‐638 mimic and substantially down‐regulated by miRNA‐638 inhibitor (Figure 3B). Second, overexpression of miRNA‐638 markedly inhibited osteogenic differentiation of hAVICs as indicated by ALP activity (Figure 3E,F) and expression of the osteogenic transcription factors Sp7 (Figure 5A,B) and osteoblast markers ALP and IBSP (Figure 3C, D and G). In contrast, low expression of miRNA‐638 significantly stimulated osteogenic differentiation which was elaborated by ALP activity (Figure 3E,F) and expression of Sp7 (Figure 5A,B), ALP and IBSP (Figure 3C, D and H).

### 3.5 MiRNA‐638 directly targets Sp7

**Figure 4:**
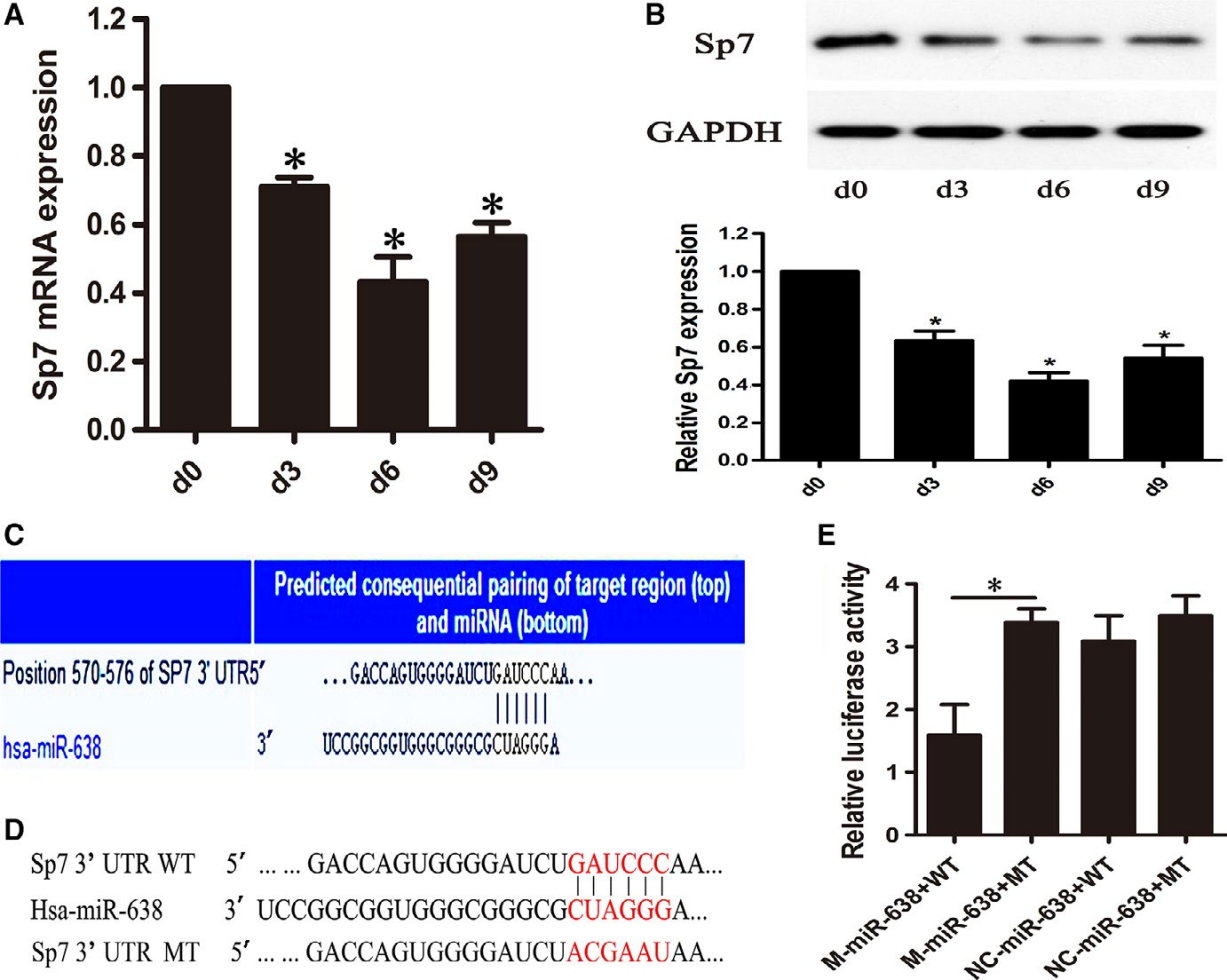
miRNA‐638 directly targets Sp7. A and B, mRNA and protein expression levles of Sp7 were determined by qRT‐PCR and western blotting at different time points (0, 3, 6 and 9 days) during osteogenic differentiation of hAVICs. The data, normalized to GAPDH, are averages of three independent experiments (mean ± SD). *P < 0.05. C, The single binding sites of miRNA‐638 (unshaded) in the 3UTR of Sp7 was predicted by TargetScan software. D, Schematic of putative miRNA‐638 target site in human Sp7 3UTR and the corresponding mutant nucleotides were coloured red. E, Dual‐luciferase assay after transfection of M‐miR‐638, NC‐miR‐638 with wide‐type Sp7 3UTR or mutant Sp7 3UTR. The data represent the mean ± SD of three independent experiments. *P < 0.05s

**Figure 5:**
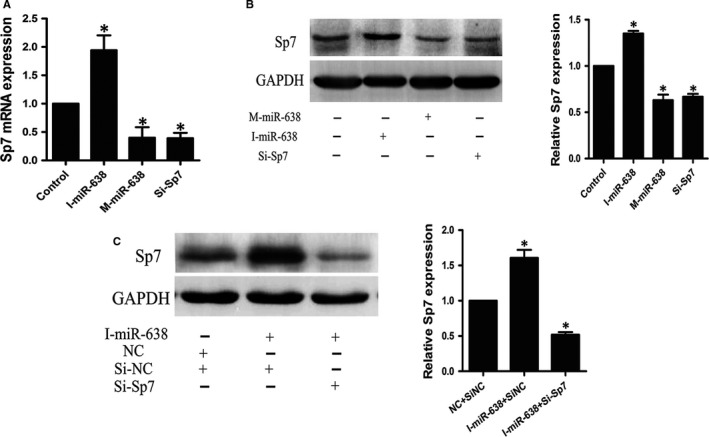
Regulation of Sp7 by miRNA‐638 during osteogenic differentiation of hAVICs. A, The mRNA expression of Sp7 after miRNA‐638 overexpression or downexpression at day 7 was analysed by qRT‐PCR. The data, normalized to GAPDH, are averages of three independent experiments (mean ± SD). *P < 0.05. B, Western blotting results showed the protein expression of Sp7 at day 14 after osteogenic differentiation of hAVICs transfected with miRNA‐638 mimic or inhibitor. The data, normalized to GAPDH, are averages of three independent experiments (mean ± SD). *P < 0.05. C, Western blotting results further confirmed Sp7 knockdown could block the effect of miRNA‐638 inhibitor during osteogenic differentiation of hAVICs. The data, normalized to GAPDH, are averages of three independent experiments (mean ± SD). *P < 0.05

To reveal the molecular mechanism by which miRNA‐638 regulates osteogenic differentiation of hAVICs, TargetScan was used to predict potential target genes of miRNA‐638. Among these candidates, Sp7 was found to contain a specific miRNA‐638 binding sites in its 3UTR (Figure 4C). During induction of osteogenic differentiation, Sp7 mRNA and protein levels decreased gradually until reaching a peak at day 6, then subsequently increased gradually (Figure 4A,B) in an inverse trend from that of miRNA‐638 levels (Figure 3A). Next, we studied whether miRNA‐638 regulates Sp7 expression during osteogenic differentiation of hAVICs. Our results showed that Sp7 mRNA and protein levels were significantly decreased by overexpression of miRNA‐638. On the contrary, Sp7 mRNA and protein levels were markedly increased by reduction in miRNA‐638 (Figure 5A,B). These data suggested that miRNA‐638 attenuated Sp7 expression during osteogenic differentiation of hAVICs.

To further confirm whether miRNA‐638 directly targets Sp7, we constructed dual‐luciferase reporters containing either wild‐type (WT) or mutant (MT) Sp7 3UTR (Figure 4D). The results verified that miRNA‐638 mimic significantly repressed luciferase activity when cotransfected with reporter containing WT Sp7 3UTR but not MT Sp7 3UTR (Figure 4E). These results indicate that miRNA‐638 directly attenuated Sp7 expression through directly binding with Sp7 3UTR.

### 3.6 Sp7 down‐regulation inhibits osteogenic differentiation of hAVICs

To investigate the functional effect of Sp7 on osteogenic differentiation of hAVICs, we suppressed Sp7 expression by transfecting hAVICs with SiRNA against Sp7 (Si‐Sp7). As shown in Figure 5A,B, both mRNA and protein levels of Sp7 were significantly decreased by Si‐Sp7. Sp7 down‐regulation inhibits osteogenic differentiation of hAVICs, indicated by IBSP and ALP mRNA expression (Figure 3C,D) and ALP activity (Figure 3E,F).

### 3.7 Sp7 knockdown could block the effect of miRNA‐638 during osteogenic differentiation of hAVICs

To confirm that the function of miRNA‐638 during osteogenic differentiation of hAVICs is mediated by repressing Sp7, we transfected miRNA‐638 inhibitors into hAVICs after Sp7 knockdown, and then proceeded with osteogenic differentiation. As is shown by western blotting (Figure 5C), miRNA‐638 inhibitors could accelerate osteogenic differentiation in Si‐Sp7 negative control (Si‐NC) group, but differentiation in the presence of inhibitors is abolished after Sp7 knockdown. These results demonstrated that deletion of Sp7 could block the effect of miRNA‐638 inhibitors, further indicating that miRNA‐638 regulates osteogenic differentiation of hAVICs through targeting Sp7.

## 4 DISCUSSION

Calcific aortic valve disease is one of the cardiovascular diseases which causing significant morbidity and mortality, especially in the elderly, and calcification plays an important role in the pathogenesis of this disease.^1^, ^38^ However, there are still no effective pharmacological treatments to prevent or treat this disease. Osteogenic differentiation of hAVICs has been confirmed to be closely associated with the pathological process of CAVD.^1^, ^9^, ^38^, ^39^, ^40^ Therefore, investigating osteogenic differentiation of hAVICs may lead to a better understanding of the pathogenesis of CAVD and improve treatment options. Recently, mounting evidence has shown that miRNAs could play a vital role in osteogenic differentiation of hAVICs.

In this study, we first screened out the differentially expressed miRNAs in CAVD pathogenesis using miRNA microarray assay. Then we further investigated the role of the differentially expressed miRNA‐638 on the pathogenesis of CAVD. At last, we successfully identified miRNA‐638 as a negative regulator of hAVICs osteogenic differentiation. Our data showed that miRNA‐638 was up‐regulated during osteogenic differentiation of hAVICs. Overexpression of miRNA‐638 inhibited osteogenic differentiation, whereas inhibition of miRNA‐638 function enhanced the osteogenic potential of these cells. In addition, we also demonstrated miRNA‐638 was significantly up‐regulated in calcific aortic valves compared with non‐calcific valves by miRNA microarray assay and qRT‐PCR results. These results suggested that miRNA‐638 could act as a protective role in the pathogenesis of CAVD. That is to say, miRNA‐638 could significantly inhibit the pathological process of CAVD. In conclusion, the schematic diagram depicting the mechanism underlying aortic valve calcification in this study was shown in Figure 3I. In the previous study, we have proved that miRNA‐449c‐5p could markedly promote the pathogenesis of this disease.^1^ These two studies indicated that multiple miRNAs may participate in the pathogenesis of CAVD. They might act as either a positive regulator or a negative regulator during the course of this disease and maintained a dynamic balance. Once this balance is disrupted, disorders such as CAVD may occur.

MiRNAs are a wide family of evolutionarily conserved, small non‐coding RNAs that play important regulatory roles by targeting mRNAs for cleavage or translational repression.^41^ Multiple previous studies have confirmed that miRNAs could function as important modulators in the pathogenetic process of many cardiovascular disorders.^42^, ^43^ Moreover, recent studies have also validated the crucial role of miRNAs during the pathogenesis of cardiovascular calcification. Cui et al Confirmed miRNA‐204 as a central regulator of vascular smooth cell calcification in vitro and in vivo by targeting Runx2.^44^ MiRNA‐141 was identified to inhibit the osteogenic differentiation of porcine VICs through a BMP‐dependent pathway.^40^ In contrast to previous studies, our study firstly used miRNA microarray assay to explore the real miRNAs involved in CAVD pathogenesis. This may make our results more representative of the actual pathological process of CAVD. Finally, miRNA‐638 was found and confirmed to negatively regulate the osteogenic differentiation of hAVICs.

miRNA‐638 was expressed in human and non‐human primates. In previous studies, dysregulation of miRNA‐638 had been described as a cohort of human tumours, including non‐small cell lung cancer,^45^ gastric cancer,^46^ breast cancer^47^ and colorectal carcinoma.^48^ These studies indicated that miRNA‐638 may participate in carcinogenesis and metastasis. For example miRNA‐638 inhibits cell growth and tubule formation by suppressing VEGF in human Ewing sarcoma cells.^49^ Another study validated miRNA‐638 could inhibit cell proliferation by targeting phospholipase D1 in human gastric carcinoma.^50^ In this study, based on preliminary research results, we successfully revealed the important role of miRNA‐638 in osteogenic differentiation of hAVICs. This study further expanded our knowledge of the function of miRNA‐638 on disease occurrence and progress.

To further elucidate the molecular mechanism by which miRNA‐638 regulates osteogenic differentiation of hAVICs, a search with TargetScan 7.2 showed that miRNA‐638 was partially complementary to a site in the 3UTR of Sp7. Expression of Sp7 increased during osteogenesis of hAVICs, which was inverse of miRNA‐638 expression. Furthermore, miRNA‐638 overexpression resulted in down‐regulation of Sp7 at the protein level, whereas functional inhibition of miRNA‐638 led to up‐regulation of Sp7, indicating that Sp7 was regulated by miRNA‐638 during osteogenic differentiation of hAVICs. Moreover, a dual luciferase reporter assay identified Sp7 as a direct target of miRNA‐638.

Sp7, which was first discovered by Nakashima et al in 2002, belongs to the specificity protein (Sp7) family. It is a zinc‐finger‐containing transcription factor which is essential for bone formation and osteogenic differentiation.^36^ In humans, the Sp7 gene, like its murine orthologue, showed osteoblast‐specific expression in vivo.^51^ To date, thousands of studies, both in vitro and in vivo, have demonstrated its vital roles and mechanisms during the above processes.^52^, ^53^, ^54^, ^55^ Riko et al have proved that Sp7 regulates calcification and degradation of chondrogenic matrices through MMP13 expression in association with Runx2 during endochondral ossification.^56^ Young Jae Moon et al confirmed that Sp7 regulated corticalization for longitudinal bone growth via integrin β3 expression in vivo.^54^ In Sp7‐null embryos, cartilage was formed normally, but the embryos totally lacked bone formation.^55^ In recent years, the co‐function of miRNAs and Sp7 during bone formation and osteogenic differentiation has also been investigated. miRNA‐637 was identified to maintain the balance between adipocytes and osteoblasts by directly targeting Sp7.^37^ miRNA‐143 and miRNA‐145 could inhibit osteogenic differentiation by targeting Sp7 and form a feedback loop with KLF4 and Sp7 in odontoblasts.^57^, ^58^ In our study, we demonstrated that miRNA‐638 inhibits osteogenesis of hAVICs by targeting Sp7.

We also found that down‐regulation of Sp7 suppressed osteogenic differentiation, similar to the effect of miRNA‐638 overexpression. Moreover, effects of miRNA‐638 inhibitor on osteogenic differentiation of hAVICs could be reversed by Sp7 SiRNA. These results provided evidence that miRNA‐638 inhibited osteogenic differentiation of hAVICs by negatively regulating Sp7.

In conclusion, we demonstrated that miRNA‐638 negatively regulated osteogenic differentiation of hAVICs by directly targeting Sp7. As far as we know, this is the first report to study the regulatory role of miRNA‐638 for human aortic valve calcification. Our study indicated that miRNA‐638 and Sp7 might be potential therapeutic targets for the management of CAVD.

## CONFLICT OF INTEREST

The authors declare no conflicts of interest.

## Data Availability

The data that support the findings of this study are available from the corresponding author upon reasonable request.
